# Characterization of m^6^A-regulated targets and immune cells in dental caries: insights from multi-omics analysis

**DOI:** 10.1186/s40001-025-03514-2

**Published:** 2025-12-24

**Authors:** Xi Zhou, Cheng Shi, Kang Li, Siyue Yao, Lan Ma, Yelin Mao, Lingling Jiang, Pengfei Jiao

**Affiliations:** 1 The Affiliated Stomatology Hospital of Suzhou Vocational Health College, Suzhou, China; 2 Changsha Stomatological Hospital, Changsha, China; 3 School of Stomatology, Hunan University of Chinese Medicine, Changsha, China; 4 Nanjing Municipal Center for Disease Control and Prevention, Nanjing, China; 5 The Affiliated Stomatology Hospital of Nanjing Medical University, Nanjing, China; 6 State Key Laboratory Cultivation Base of Research, Prevention and Treatment for Oral Diseases, Nanjing, China; 7 Jiangsu Province Engineering Research Center of Stomatological Translational Medicine, Nanjing, China

**Keywords:** Dental caries, Genome-wide association studies, *N*^6^-Methyladenosine, Single-cell RNA sequencing, Mendelian randomization, Immune cells

## Abstract

**Background:**

Dental caries is one of the most common oral diseases worldwide. *N*^6^-Methyladenosine (m^6^A), a type of RNA modification, plays an important role in various biological processes and cellular responses. This study aimed to investigate the causal relationship between specific genetic loci related to m^6^A and dental caries.

**Methods:**

Caries-associated genetic loci and signaling pathways were explored by integrating publicly available single-nucleotide polymorphism (SNP) association data, m^6^A quantitative trait locus (m^6^A-QTL) data from genome-wide association studies (GWAS), and single-cell RNA sequencing data derived from the dental pulp of healthy individuals and patients with dental caries. Mendelian randomization (MR) was also used to explore the causal relationship between m^6^A-related genetic variants and dental caries.

**Results:**

Among 29,797 m^6^A-SNPs analyzed, four reached genome-wide significance (*P* < 5.0E-05), with rs3829225 showing the most significant association (*P* = 1.23E-06). The rs2259956 variant demonstrated significant *cis*-expression quantitative trait locus (cis-eQTL) effects on *ABHD12* expression (*z* score = 19.072, *P* = 4.35E-81) and was predicted to enhance m^6^A modification (score change: 0.588 to 0.595). Single-cell RNA sequencing of 53,595 cells revealed that dendritic cells mainly existed in the carious pulp and showed the highest fold-change, with *ABHD12* significantly upregulated in carious samples. Gene set enrichment analysis identified oxidative phosphorylation as the top enriched pathway (FDR < 0.05). Two-sample MR analysis (161,113 cases, 216,164 controls) revealed causal relationships between 15 immune cell traits and dental caries, with CD62L^−^dendritic cells increasing dental caries risk (OR = 1.019, 95% CI 1.004–1.035, *P* = 1.6E-02) and plasmacytoid dendritic cells showing protective effects (OR = 0.978, 95% CI 0.965–0.991, *P* = 1.0E-03).

**Conclusions:**

This study presents new evidence characterizing m^6^A-related SNPs in increasing dental caries susceptibility and provides a single-cell transcriptomic profiling of dental caries with insights into the relationship between dental caries and immune cells.

**Supplementary Information:**

The online version contains supplementary material available at 10.1186/s40001-025-03514-2.

## Introduction

Dental caries is one of the most common oral diseases, characterized by a high prevalence, low treatment rate, and widespread and persistent harm to human health. According to the Global Burden of Disease Study in 2019, approximately 64.6 and 62.9 million people suffer from dental caries in their permanent and deciduous teeth respectively [1]. According to Keyes’ triad theory, dental caries results from the interaction of three primary factors: host (tooth and saliva), microflora (cariogenic bacteria), and substrate (diet), over time. Fejerskov’s ecological plaque hypothesis further emphasizes the dynamic nature of this process, where environmental changes can shift the oral microbiome balance.

Over the past decade, genome-wide association studies (GWAS) have identified numerous dental caries-specific genetic variations in functional genomic sequences [2]. Genetic variation at rs7738851 on *NEDD9* influenced susceptibility to dental caries in permanent teeth, whereas rs1594318 within *ALLC* on 2p25 was associated with dental caries in primary teeth [3]. Despite evidence of genetics contributing to dental caries, only a few genetic loci have been explored.

As a widely distributed and abundant internal RNA modification in eukaryotes, *N*^6^-methyladenosine (m^6^A) modification plays an important role in various biological processes (BP) and cellular responses [4]. In the process of m^6^A modification, methyltransferases such as METTL3/METTL14 can recognize and methylate the DRACH motif (where D = A, G, U; R = A, G; H = A, C, U) in RNA sequences, which is referred to as m^6^A sites. However, when a mutation occurs at the base of the DRACH motif, m^6^A regulators fail to correctly recognize these motifs, and the m^6^A modification process is thus affected. Therefore, *N*^6^-methyladenosine single-nucleotide polymorphisms (m^6^A-SNPs) are regarded as a key type of genetic variation that influences the development of numerous diseases [5, 6]. For example, the mutation of rs11266744 altered the m^6^A modification of *CCRL2*, regulated the expression of the local gene *CCRL2*, and thereby participated in the pathogenesis of oral ulcers [7]. However, significant gaps remain in our understanding of how m^6^A-related SNPs interact with the caries risk factors.

Previous GWAS studies have primarily focused on coding variants, with limited investigation of RNA modification-associated polymorphisms. For instance, while Shungin et al. identified several dental caries-associated loci, none specifically examined m^6^A modifications [2]. Similarly, studies by Haworth et al*.* on childhood dental caries traits did not explore epigenetic RNA modifications despite growing evidence of their importance in immune responses and tissue homeostasis [3]. The role of genetic polymorphisms, particularly m^6^A-SNPs, in modulating individual susceptibility within this multifactorial framework remains largely unexplored. Understanding how genetic variants interact with these classical factors is crucial for developing personalized prevention strategies.

In addition to the aforementioned mechanisms, distinguishing between correlation and causation is essential. Mendelian randomization (MR) is an observational data technique that assesses whether genetic predictors of a given exposure are associated with an outcome [8]. For example, causal relationships between five metabolites and dental traits were inferred, and increases in their levels were genetically associated with a lower risk of bleeding gums [9]. Further, a strong association was identified between the quality of gut microbiota and oral ulcers [10]. Chmilewsky et al. reported that the complement system was activated at the injured site of human carious teeth and may play an important role in dental-pulp regeneration [11]. Another study further determined the active fragment of the complement C5a receptor in dental caries injury [12]. These results suggested that immune cells may play an important role in caries progression.

**Fig. 1:**
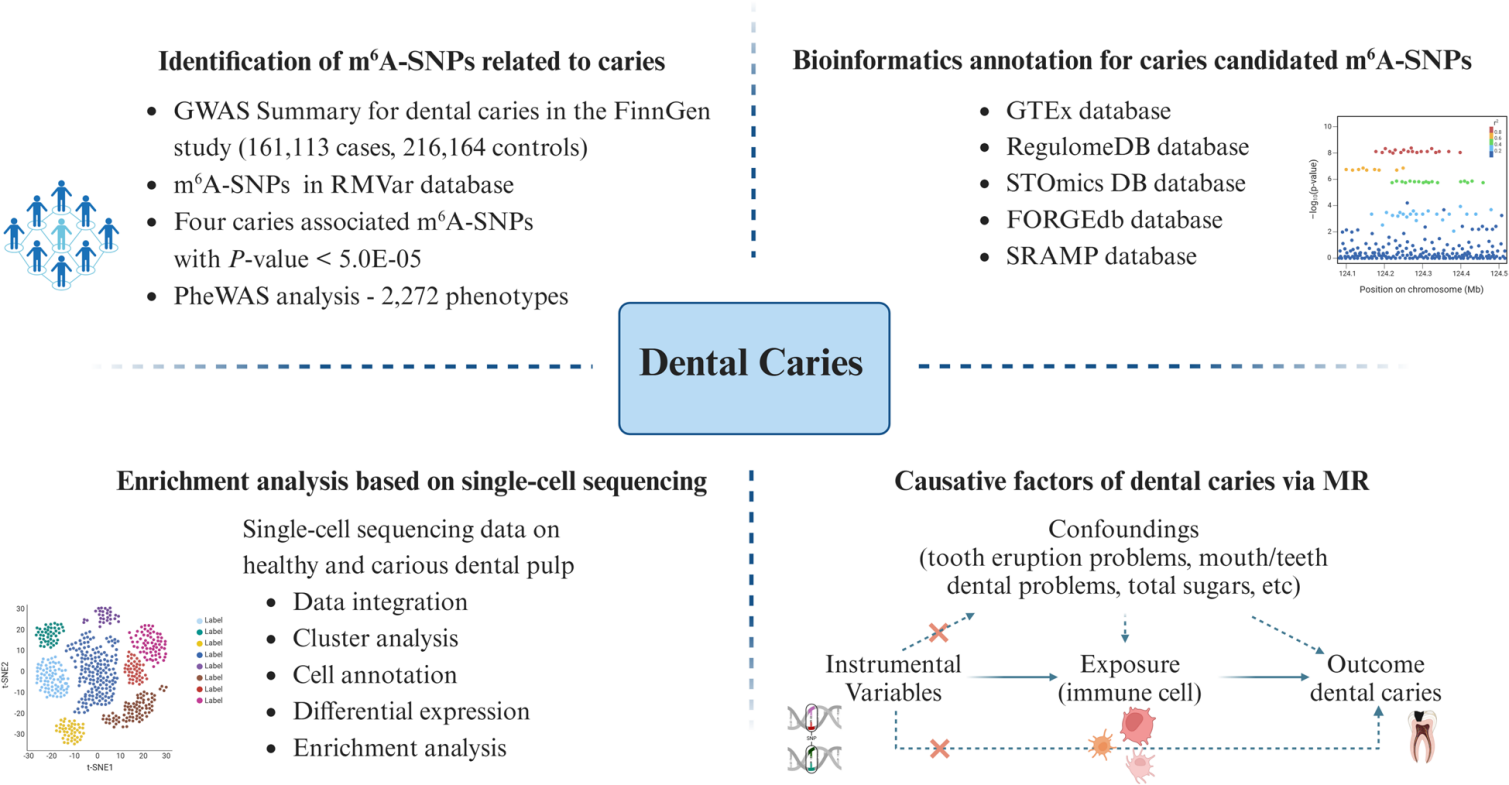
Flow chart of the study design

This study systematically evaluated the impact of m^6^A-SNPs on dental caries by integrating publicly available SNP association data from GWAS and m^6^A quantitative trait loci (QTL) data. By analyzing the data on human dental pulp from healthy individuals and those with dental caries, the cell types and functions associated with dental caries susceptibility were determined. Additionally, this study assessed the cellular expression of m^6^A-SNP candidate genes linked to the occurrence of dental caries. Finally, a two-sample MR design was used to determine immune cell involvement in dental caries risk, with a schematic overview of the study design provided in Fig. 1. Collectively, this study first uncovers new evidence characterizing m^6^A-related SNPs in increasing dental caries susceptibility and provides a single-cell transcriptomic profile of dental caries.

## Materials and methods

### Identification of m^6^A-SNPs in human dental caries

To identify dental caries-associated m^6^A-SNPs, relevant GWAS statistics and m^6^A-QTL data were integrated. GWAS summary data for dental caries were downloaded from the FinnGen Consortium (accessed on 1 June 2024, https://r9.finngen.fi/pheno/K11_CARIES_1_ONLYAVO), encompassing 161,113 cases and 216,164 controls. The Finnish population was selected due to the availability of large-scale, well-phenotyped GWAS data from the FinnGen Consortium. This dataset features well-defined phenotype definitions using ICD-10 codes with clinical validation, rigorous quality control (including Hardy–Weinberg equilibrium testing, call rate filtering, and population stratification correction), and publicly accessible summary statistics. However, we acknowledge that this choice limits the ethnic diversity of our genetic analysis. A list of human m^6^A-SNPs was obtained from the publicly available RMVar database (accessed June 2024, https://rmvar.renlab.org/index.html), which catalogs 1,401,814 m^6^A-associated variants with high, medium, and low confidence prediction stratification based on experimental validation from multiple m^6^A-seq technologies (MeRIP-seq, m^6^A-CLIP-seq), and maintains regular updates with standardized genome coordinates (GRCh38). The FinnGen GWAS and m^6^A-QTL data from the RMVar database were both aligned to GRCh38. The database provided 132,428 high-confidence, 299,027 medium-confidence, and 290,370 low-confidence levels for human m^6^A-SNP prediction. The qqman (v.0.1.8, RRID: SCR_024293) package was used to generate a Manhattan plot for dental caries-related m^6^A-SNPs.

### Phenome-wide association analysis

A phenome-wide association analysis (PheWAS) was conducted to investigate possible associations between dental caries‐associated m^6^A‐SNPs and 2,268 binary and 3 quantitative disease endpoints. Statistically independent SNPs were grouped as genetic instruments, and outcome phenotypes were selected using FinnGen (https://r9.finngen.fi/). This analysis was performed directly using the FinnGen website, which provides built-in tools for visualizing association data across multiple phenotypes. The false discovery rate (FDR) derived via the Benjamini–Hochberg procedure was used to account for type 1 error inflation due to multiple testing.

### In silico analysis of candidate m^6^A-SNPs and their parental genes

The RegulomeDB (RRID: SCR_017905) [13] and FORGEdb [14] online bioinformatics tools were used to identify the potential function of m^6^A-SNPs associated with dental caries. In general, with more supporting data, a higher RegulomeDB score indicates the likelihood of being functional, thus receiving a higher score (with 1 being higher and 7 being lower; i.e., 1a–f were all scored as 1). FORGEdb scores range between 0 and 10 and are calculated using different regulatory DNA datasets. To evaluate the candidate gene expression signatures in single cells, data were obtained from the spatiotemporal transcriptomic atlases of mouse organogenesis (https://db.cngb.org/stomics/mosta). Additionally, a dynamic spatiotemporal transcriptomic atlas (https://db.cngb.org/stomics/zesta/) of zebrafish embryogenesis at 48 and 72 h post-fertilization (hpf) was utilized to explore target gene expression.

### Prediction of m^6^A modification near candidate m^6^A-SNPs

A sequence-based RNA adenosine methylation site predictor (SRAMP, http://www.cuilab.cn/sramp/, RRID: SCR_024500) was used to predict whether dental caries-associated m^6^A-SNPs affect the surrounding m^6^A modifications. The online m^6^A modification prediction tool classified m^6^A sites with very high, high, moderate, or low confidence according to thresholds of 99%, 95%, 90%, and 85% specificity, respectively. By inputting genomic or cDNA sequences containing reference and altered alleles, the prediction score and confidence level of m^6^A modification sites were obtained. To identify statistically significant m^6^A peaks, such peaks were derived from Methylated RNA Immunoprecipitation Sequencing (MeRIP-seq) data [19] and m^6^A-label-seq data [20] (GSE131316 and CRA001315).

### Expression QTL analysis and colocalization of dental caries‐associated m^6^A‐SNPs

Expression QTL (eQTL) analysis was used to assess whether m^6^A-SNPs influenced their corresponding host gene expression. The eQTL and splicing QTL (sQTL) data of 54 human tissue/cell types were downloaded from GTEx v.8 (accessed June 2024, RRID: SCR_013042), and information on other types of molecular trait QTL (xQTL) was collected from QTLbase v.2.2. Further, to increase the power of detecting cis-eQTL associations in whole blood, cis-eQTL summary statistics were obtained from eQTLGen (accessed July 2024, https://www.eqtlgen.org). LocusCompare [15] was used to visualize the GWAS and eQTL colocalization events.

### Single-cell transcriptome sequencing analysis

Single-cell transcriptome profile data for healthy (GSE164157) and carious pulp (GSE251953) were obtained from the Gene Expression Omnibus database (RRID: SCR_005012), both of which utilized the 10 × Genomics platform to ensure methodological consistency and contained high-quality sequencing data of over 10,000 cells with comprehensive cell type representation. To guarantee the quality of data integration, batch correction methods from the Harmony package (v.1.2.0, RRID: SCR_022206) were applied alongside quantitative assessment. The Seurat (v.5.1.0, RRID: SCR_007322) package was used to generate the object and to filter out poor-quality cells. Using standard data preprocessing, the percentages of gene numbers, cell counts, and mitochondrial sequencing counts were calculated. Then, genes detected in fewer than three cells were excluded, and cells with fewer than 200 detected genes were disregarded [16]. Corrected normalized data metrics were applied to the standard analysis. Unsupervised clustering using Uniform Manifold Approximation and Projection (UMAP) was used to identify six single-cell clusters. SingleR (v.2.0.0, RRID: SCR_023120) was used to identify the cell types in each cluster.

### Enrichment analyses

To identify differentially expressed genes, pairwise comparisons of individual clusters against all other clusters were performed using the FindAllMarkers function (settings: min.pct = 0.25, thresh.use = 0.25) in Seurat. Pathway enrichment analysis of the differentially expressed genes was performed using the clusterProfiler (v.4.6.2, RRID: SCR_016884) package to determine which biological functions or processes were significantly enriched based on Gene Ontology (GO) and Gene Set Enrichment Analysis (GSEA).

### Two-sample Mendelian randomization analysis

To investigate the causal relationships between immunocytes and dental caries, two-sample MR analyses were performed. This approach uses genetic variants as instrumental variables (IVs) to infer causality while minimizing confounding and reverse causation [17]. The validity of MR relies on three core assumptions: 1. Relevance assumption: Genetic instruments must be robustly associated with the exposure. This study selected SNPs at *P* < 1.00E-05 and calculated F statistics for each instrument, with F > 10 indicating sufficient strength to avoid weak instrument bias. 2. Independence assumption: Genetic instruments must not be associated with confounders of the exposure–outcome relationship. Additionally, this analysis addressed this through: (a) using germline genetic variants that are randomly allocated at conception; (b) performing linkage disequilibrium (LD)-clumping (*r*^2^ < 0.001, window = 10,000 kb) to ensure independence between instruments; (c) checking for population stratification effects. 3. Exclusion restriction assumption: Genetic instruments must affect the outcome only through the exposure.

Comprehensive sensitivity analysis was performed as follows: (a) Heterogeneity assessment: Cochran’s Q statistic was used to test for heterogeneity among SNP-specific causal estimates; significant heterogeneity (*P* < 5.0E-02) indicates a potential violation of MR assumptions. (b) Leave-one-out analysis: This involved the systematic removal of each SNP to identify variants with influential effects on causal estimates. (c) Funnel plot inspection: Asymmetry in the funnel plot was visually assessed, with such asymmetry indicative of directional pleiotropy. (d) Multiple MR methods: Estimates derived from different MR approaches, including inverse-variance weighted (IVW, primary method), MR-Egger, weighted median, and weighted mode, were compared to evaluate result consistency.

The GWAS catalog (GCST90001391-GCST90002121, accessed June 2024) provides comprehensive immunological data from 3757 Europeans comprising 731 immunophenotypes [18]. These immunophenotypes encompassed: absolute cell counts (*n* = 118 traits), including total leukocytes, lymphocytes, monocytes, neutrophils, eosinophils, and basophils; cell percentages (*n* = 192 traits), indicating the proportions of major immune cell types and subsets; detailed T cell phenotypes (*n* = 211 traits), comprising CD4^+^, CD8^+^, regulatory T cells (T_regs_), memory, naïve, and activated subsets; B cell phenotypes (*n* = 124 traits), including IgD/CD38-defined subsets, memory B cells, and plasmablasts; and myeloid cell phenotypes (*n* = 86 traits), comprising dendritic cell (DC) subsets, (plasmacytoid, conventional, CD62L^+/−^), monocyte subsets, and activation markers. MR analysis was conducted using the TwoSampleMR (v.0.6.4, RRID: SCR_019010) package. The resulting information was extracted from the IEU OpenGWAS or FinnGen databases, and relationships between SNPs that satisfied the hypotheses were then obtained.

### Statistical analysis

Continuous variables were first tested for normality using the Shapiro–Wilk test and visual inspection of Q–Q plots. Variables with normal distributions were presented as mean ± standard deviation (SD), whereas non-normally distributed variables were presented as median with interquartile range (IQR: Q1–Q3). Statistical analysis was performed using SPSS (v.20.0; SPSS, Inc., Chicago, IL, USA, RRID: SCR_016479), and figures were plotted using GraphPad Prism v.7.0 (GraphPad Software, La Jolla, CA, USA, RRID: SCR_002798). All bioinformatics analyses were conducted using R statistical software (v.4.3.1; R Foundation for Statistical Computing, Vienna, Austria; https://www.R-project.org/, RRID: SCR_001905). The following R packages were utilized: qqman for Manhattan plot generation, Seurat for single-cell RNA sequencing analysis, Harmony for batch effect correction in single-cell RNA seq integration, SingleR for automated cell type annotation, clusterProfiler for GO and GSEA pathway enrichment analyses, TwoSampleMR for MR analyses, tidyverse (v.2.0.0, RRID: SCR_019186) for data manipulation and visualization, and ggplot2 (v.3.4.2, RRID: SCR_014601) for additional data visualization. In this study, we analyzed dental caries as a binary outcome (presence vs. absence of caries) using logistic regression. For continuous traits, such as immune cell proportions, linear regression was employed to assess the relationship between genetic variants and the respective traits. The models were adjusted for potential confounders, and statistical significance was determined using a threshold of *P* < 5.0E-02. For MR analysis, sensitivity analysis included Cochran’s *Q* test for heterogeneity, the MR-Egger intercept test for horizontal pleiotropy, and leave-one-out analysis. All results were presented as odds ratios (OR) and 95% confidence intervals (CIs) where applicable, and were considered statistically significant at *P* < 5.0E-02.

## Results

### Identification of the association between m^6^A-SNPs and dental caries

**Fig. 2:**
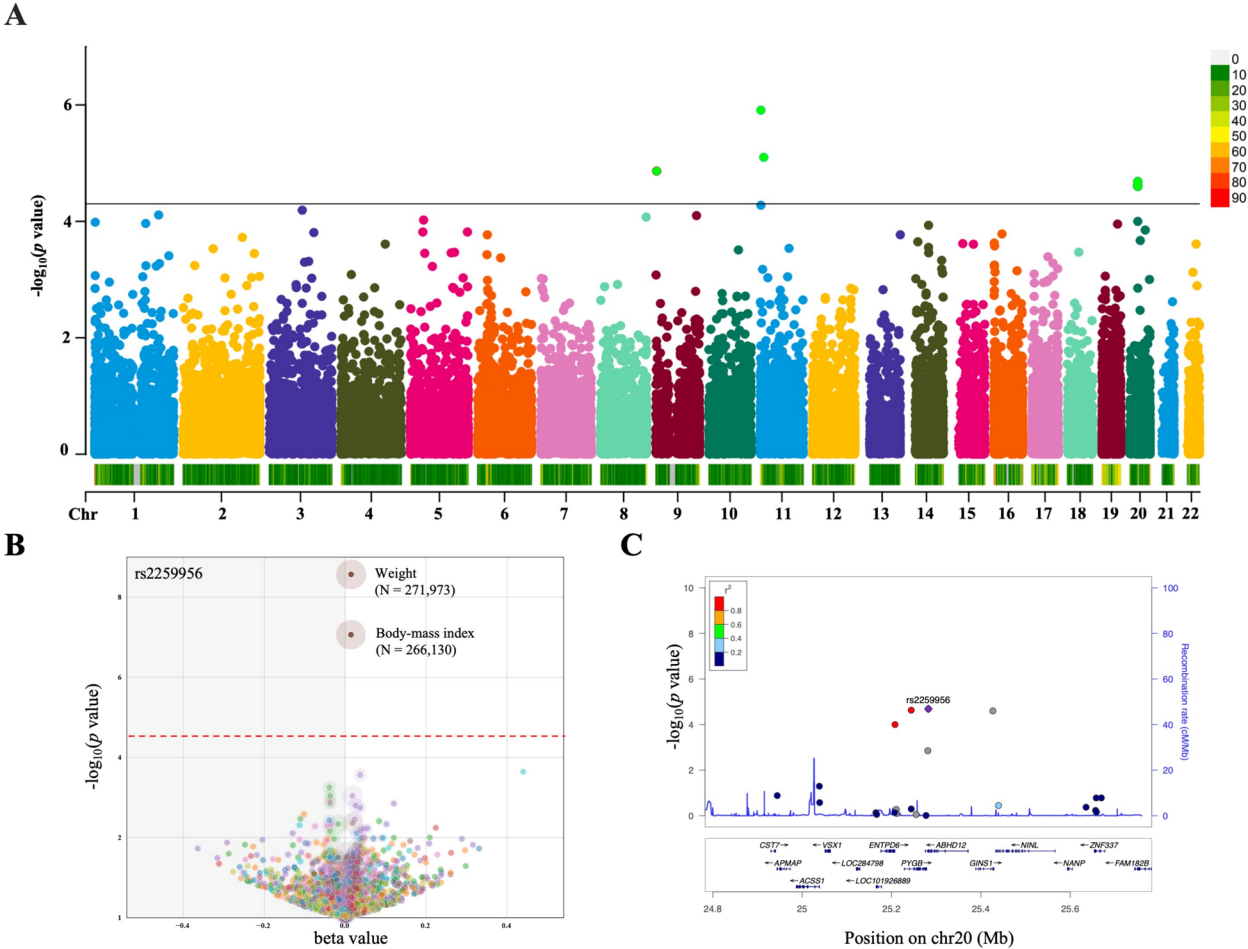
Distribution of dental caries-associated m^6^A-SNPs identified in GWAS. **A**. Manhattan plot of 29,797 m^6^A-SNPs in the context of dental caries. The line shows m^6^A-SNPs reaching genome-wide significance (*P* < 5.0E-05). Colors indicate different chromosomes. Four significant m^6^A-SNPs are above this threshold. **B**. PheWAS volcano plot for rs2259956 across 2270 phenotypes (FinnGen, *N* = 377,277). This figure represents 2270 phenotypes tested for association with the SNP. The upper-red line indicates Bonferroni correction *P* = 2.2E-05 (false discovery rate = 0.1 for entire PheWAS). **C**. Regional association plot for rs2259956. Upper panel: SNP associations with rs2259956 highlighted in red. Color scale represents linkage disequilibrium (LD) with rs2259956. Lower panel: recombination rate and gene annotations, showing *ABHD12* as the host gene

In this study, 20,170,236 SNPs from the GWAS for dental caries and 1,401,814 m^6^A-SNPs from the RMVar database were integrated for analysis, which ultimately identified overlapping 29,797 m^6^A-SNPs (Fig. 2A). This analysis successfully identified four genome-wide significant m^6^A-SNPs associated with dental caries, representing the first evidence linking m^6^A modifications to caries susceptibility at the genomic level. All four m^6^A-SNPs reached a recommended threshold of *P* < 5.0E-05 after removing all SNPs in high LD (*r*^2^ > 0.800), and they showed strong regulatory potentials, with rs2259956-*ABHD12* emerging as the lead candidate due to its significant cis-eQTL effects and predicted functional gain in m^6^A modification (Table 1). Specifically, rs3829225 was the most significant of the dental caries-related m^6^A-SNPs (*P* = 1.23E-06). Furthermore, a PheWAS was conducted in the FinnGen study (*N* = 377,277), with associations between four individual SNPs and 2270 endpoints examined (Appendix Fig. 1). Intriguingly, the strongest PheWAS associations of rs2259956 with weight (*N* = 271,973) and body mass index (*N* = 266,130) were identified (Fig. 2B). The PheWAS results revealed pleiotropic effects of rs2259956 beyond dental caries, particularly on metabolic traits, suggesting broader health implications of this m^6^A variant. Meanwhile, the regional plot showed that rs2259956 was located in the *ABHD12* gene (Fig. 2C). Literature searches were conducted to compile the potential impacts of these four genes on human health, with relevant information summarized in Appendix Table 1.

**Table 1:** The information of four dental caries-associated m^6^A-SNPs

Variants	CHR	Position	Gene region	Ref/Alt	beta	*P*	Gene	eQTL*	RegulomeDB Rank*	FORGEdb score*	TF bound	Motifs changed	m^6^A_ID	m^6^A_Function*
rs3829225	11	1,461,621	3′-UTR	T/C	−0.02	1.23E-06	*BRSK2*	No	1d	10	Yes	Yes	RMVar_ID_170913	Functional loss
rs1340193952	11	10,693,881	2 KB of 5'UTR	C/T	−0.28	7.94E-06	*IRAG*	No	4	–	Yes	Yes	RMVar_ID_899610	Functional gain
rs10757686	9	2,787,856	16 KB of 5'UTR	A/C	0.02	1.37E-05	*PUM3*	No	1f	7	Yes	Yes	RMVar_ID_641337	Functional loss
rs2259956	20	25,301,797	intron	A/G	0.02	2.06E-05	*ABHD12*	Yes	1f	7	Yes	Yes	RMVar_ID_1203844	Functional gain

^*^Based on eQTLGen blood FDR 0.05 cis-eQTL information, eQTL influenced expression on the nearest gene

^*^RegulomeDB Rank was downloaded from RegulomeDB (https://regulomedb.org/)

^*^FORGEdb score was downloaded from FORGEdb (https://forgedb.cancer.gov/)

^*^Functional Gain indicates that the variant allele is predicted to create or enhance m^6^A modification at the site; Functional Loss indicates that the variant allele is predicted to disrupt or reduce m^6^A modification at the site. These predictions are based on sequence motif analysis and m^6^A modification probability scores from SRAMP

### Functional annotation of dental caries-associated m^6^A-SNPs

According to RegulomeDB, rs3829225 had a rank score of 1 d (i.e., eQTL/caQTL (chromatin accessibility QTL) + transcription factor (TF) binding + any motif + chromatin accessibility peak), whereas rs10757686 and rs2259956 displayed a score of 1f (i.e., eQTL/caQTL + TF binding/chromatin accessibility peak), indicating that they could have a strong regulatory function, such as in TF binding or chromatin accessibility. In contrast, rs1340193952 had a score of 4, indicating minimal binding (Table 1).

FORGEdb scores (with ranks 0–10 representing increasing regulatory evidence) were utilized to verify the genetic variants most likely to have a regulatory function. The resulting FORGEdb score for rs3829225 was 10 (highest), whereas those for rs10757686 and rs2259956 were 7, indicating a regulatory role for these two SNPs. However, rs1340193952 did not provide such evidence based on the FORGEdb records.

**Fig. 3:**
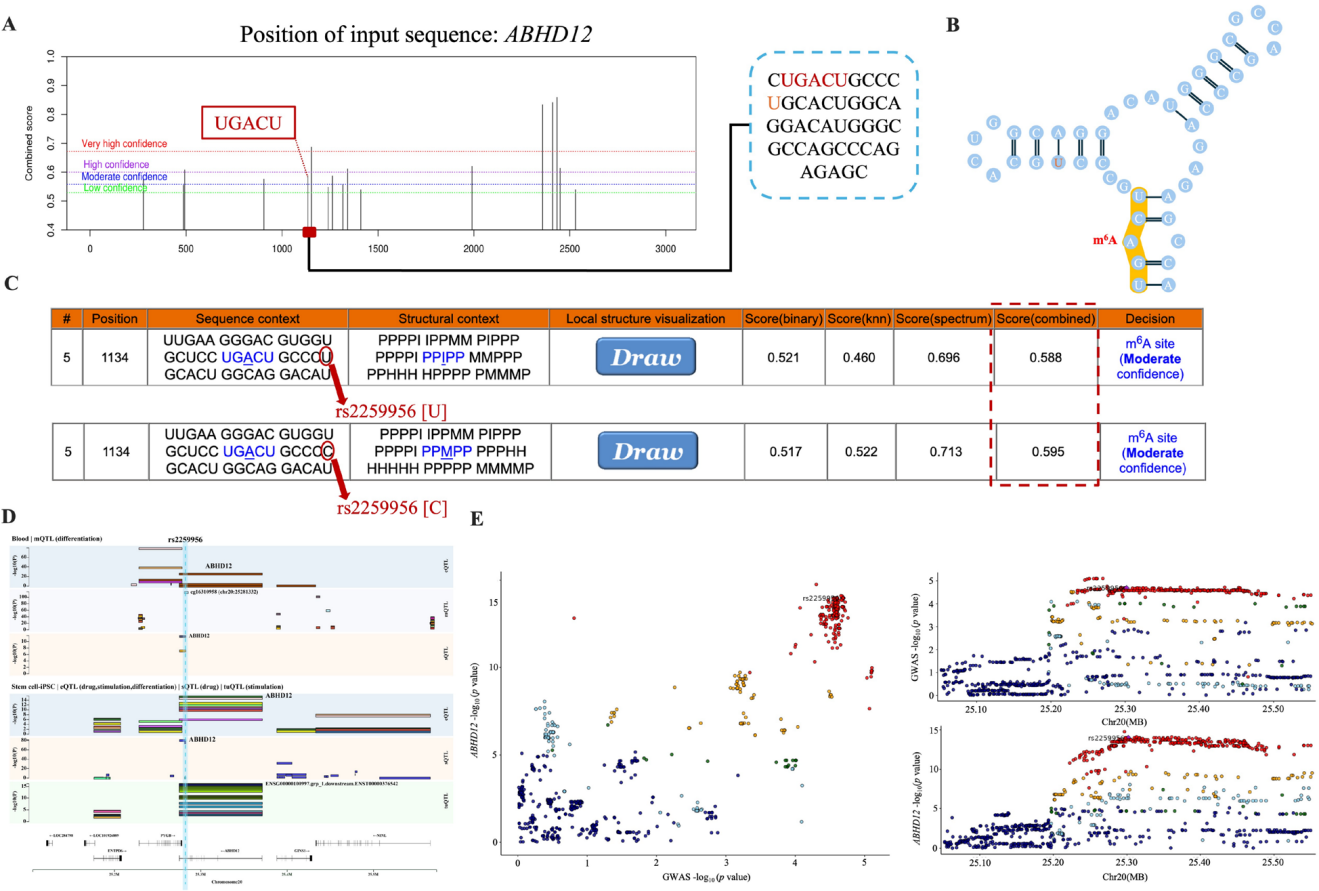
Functional annotation of dental caries-associated m^6^A-SNPs. **A**, **B**. Potential m^6^A sites predicted using SRAMP. **C**. The rs2259956 variant located 7 bp from a predicted m^6^A site annotated using the SRAMP database. **D**. eQTL analysis of rs2259956 and *ABHD12* expression genome-wide QTL mapping across tissues. QTL, quantitative trait loci. **E**. Colocalization between the GWAS signal at rs2259956 and expression QTL mapping using stem cell-iPSC

Next, to investigate whether the aforementioned dental caries-associated m^6^A-SNPs could affect m^6^A methylation sites, the genomic sequences of four genes (*BRSK2*, *IRAG*, *PUM3*, and *ABHD12*) were entered into the SRAMP predictor. The results revealed that rs2259956 in the intron of *ABHD12* was located 7 bp away from a moderately convincing predicted m^6^A-modification site, and the altered sequence increased the prediction score from 0.588 to 0.595 (Fig. 3A–C). This functional analysis provides compelling evidence that rs2259956 enhances m^6^A modification probability, establishing a direct mechanistic link between the genetic variant and epigenetic regulation. In the 2 kb region upstream of *IRAG*, rs1340193952 was 10 bp away from a moderately convincing m^6^A-modified predicted site (Appendix Fig.  2 A), and the altered sequence increased the prediction score. The m^6^A-associated variant rs3829225, with a medium confidence level, was identified from MeRIP-seq experiments (Appendix Fig. 2B). Furthermore, the credible m^6^A site rs10757686 was identified using m^6^A-label-seq (Appendix Fig.  2 C).

A thorough extrapolation was conducted on the comprehensive catalog detailing the genetic influences on gene expression, with the rs2259956 variant showing a significant association with *ABHD12* expression in induced pluripotent stem cells (Fig. 3D, E). The strong eQTL signal (*z* score = 19.072, *P* = 4.35E-81) demonstrated that rs2259956 robustly increased *ABHD12* expression, completing the pathway from genetic variation through m^6^A modification to altered gene expression. Analysis of eQTLGen Consortium data revealed that the G allele of rs2259956 was associated with increased expression of the corresponding gene, *ABHD12* (z score = 19.072, *P* = 4.35E-81). However, no evidence showed that rs3829225 affected *BRSK2*, rs1340193952 exerted an eQTL effect on *IRAG*, or rs10757686 affected *PUM3*. Therefore, these results suggested that rs2259956 may alter the m^6^A methylation level of *ABHD12* by affecting nearby m^6^A modification sites. Additionally, a comparison was conducted between the m^6^A-SNP rs2259956 and established caries-associated variants, with detailed information summarized in Online Appendix Table 2.

To explore the function of *ABHD12* in development, spatial transcriptome data from C57BL/6 mouse embryos at embryonic day 12.5 (E12.5) were analyzed. The expression of *Abhd12* was predominantly clustered in regions associated with the jaw and teeth (Appendix Fig.  3 A, B). Furthermore, single-cell transcriptome data of zebrafish embryogenesis at 48 and 72 hpf highlighted *abhd12* expression in specialized phagocytes, as well as in macrophages and neutrophils, respectively (Appendix Fig. 3 C, D).

### Integrated analyses of single-cell transcriptomes from healthy and carious pulp

**Fig. 4:**
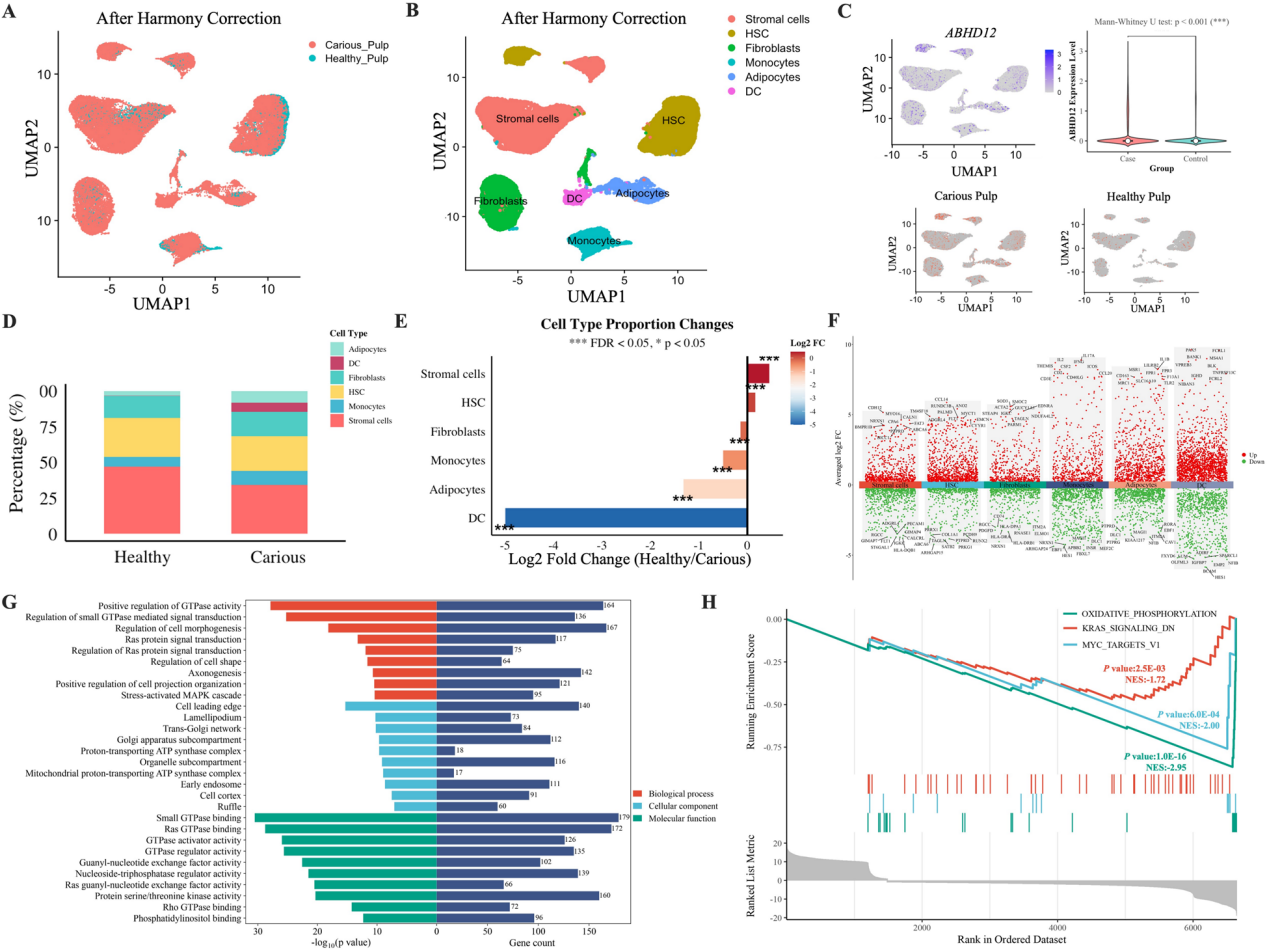
Single-cell sequencing analysis of healthy and carious pulp. **A**. Uniform manifold approximation and projection (UMAP) plot of visualization of integrated single cells after Harmony batch correction. **B**. UMAP plot of the single-cell profile colored by clusters. **C**. *ABHD12* gene expression visualization on UMAP and violin plot comparison between groups (bottom). Mann–Whitney *U* test *P* value < 1.0E-03. **D**. Stacked bar chart showing cell type proportions in healthy versus carious pulp. **E**. Log2 fold change of cell type abundances (Healthy/Carious ratio) with statistical significance indicated. ***FDR < 0.001, **FDR < 0.01, *FDR < 0.05. **F**. Differential gene expression analysis showing significantly altered genes, with red and green dots representing upregulated and downregulated genes, respectively, while an adjusted *P* value $\ge$ 1.0E-02 is indicated in black. **G**. Gene Ontology (GO) enrichment analysis of DEGs. BP is marked by red, CC is marked by blue, and MF is marked by green. The top ten terms in each category were ranked by statistical significance, with the most significant terms (lowest *P* values, corresponding to highest -log_10_*P* values) shown at the top of each bar chart. **H**. Gene set enrichment analysis (GSEA) plot identifying the three most significantly enriched hallmark pathways between healthy and carious pulp groups

To further elucidate the functional mechanism of dental caries, single-cell transcriptomes from healthy and carious pulp samples were integrated using Harmony batch correction. This approach successfully eliminated technical batch effects while preserving biological variation, as demonstrated by improved batch mixing scores and near-zero batch silhouette scores (Appendix Fig. S4A, B and Online Appendix Table 3). While bulk RNA seq could reveal overall expression differences between healthy and carious pulp tissues, single-cell RNA sequencing uniquely enabled us to: (1) identify specific cell types driving the expression changes of m^6^A-regulated genes, particularly revealing that *ABHD12* upregulation was most prominent in DCs; (2) discover the dramatic shift in immune cell populations, with DCs being predominantly present in carious but not healthy pulp; and (3) uncover cell type-specific pathway enrichments, showing that changes in oxidative phosphorylation were specifically enriched in immune cell populations. This cellular resolution was critical for establishing the mechanistic link between m^6^A modifications and immune cell-mediated caries progression, which would have been masked in bulk tissue analysis. After quality control, 53,595 cells and 24,682 genes were retained for further analysis (Appendix Fig. 4SC–G). Various cell types were clustered, identified, and visualized in UMAP plots, which demonstrated successful data merging and batch effect removal after batch correction (Fig. 4A, B). The integrated single-cell analysis revealed dramatic cellular and molecular changes in carious pulp, including significant *ABHD12* upregulation (*P* = 1.12E-143) and a striking 32-fold enrichment of DCs (from 0.20% to 6.39%), identifying DCs as central players in caries progression. Notably, *ABHD12*, which is mainly expressed in carious pulp, validates that upregulation of *ABHD12* would increase the incidence of developing dental caries (Fig. 4C and Online Appendix Table 4).

The percentage of DCs was found to account for only 0.20% of healthy pulp cells and increase to 6.39% in carious pulp, representing the highest fold-change (FC) enrichment (Log2 FC = − 4.998, adjusted *P* < 1.0E-03) (Fig. 4D, E and Online Appendix Table 5). DCs are particularly engaged in phagocytosis, suggesting a potential causal relationship between immune cells and dental caries.

Differential gene expression analysis of the upregulated and downregulated genes in each cluster was performed (Fig. 4F and Appendix Fig. 5). GO enrichment analysis of differentially expressed genes between healthy and carious pulps yielded 1,342 BP, 193 cellular components (CC), and 252 molecular functions (MF) (Appendix Table 6). The top nine of each category were sorted according to the Log *P* value from small to large, and a histogram was drawn (Fig. 4G). The top ranks belonged to the regulation of GTPase activity, positive regulation of GTPase activity, and regulation of small GTPase-mediated signal transduction enrichment in BP; cell leading edge, lamellipodium, and *trans*-Golgi network enrichment in CC; and small GTPase binding, Ras GTPase binding, and GTPase activator activity in MF. GSEA further revealed that cellular functions associated with oxidative phosphorylation (all FDR < 5.0E-02) were enriched in dental caries, indicating that dental caries incidence was increased by regulating the oxidative phosphorylation pathway (Fig. 4H and Online Appendix Table 7).

### Causal effects of immunocytes on dental caries

Based on the above findings, an MR approach was applied to resolve potential causal relationships between dental caries and immunocytes, particularly DCs. The GWAS data of 731 immunocyte phenotypes were screened for IVs; all IVs had F values greater than 10, and no weak IV bias was observed, satisfying the relevance assumption of MR.

**Fig. 5:**
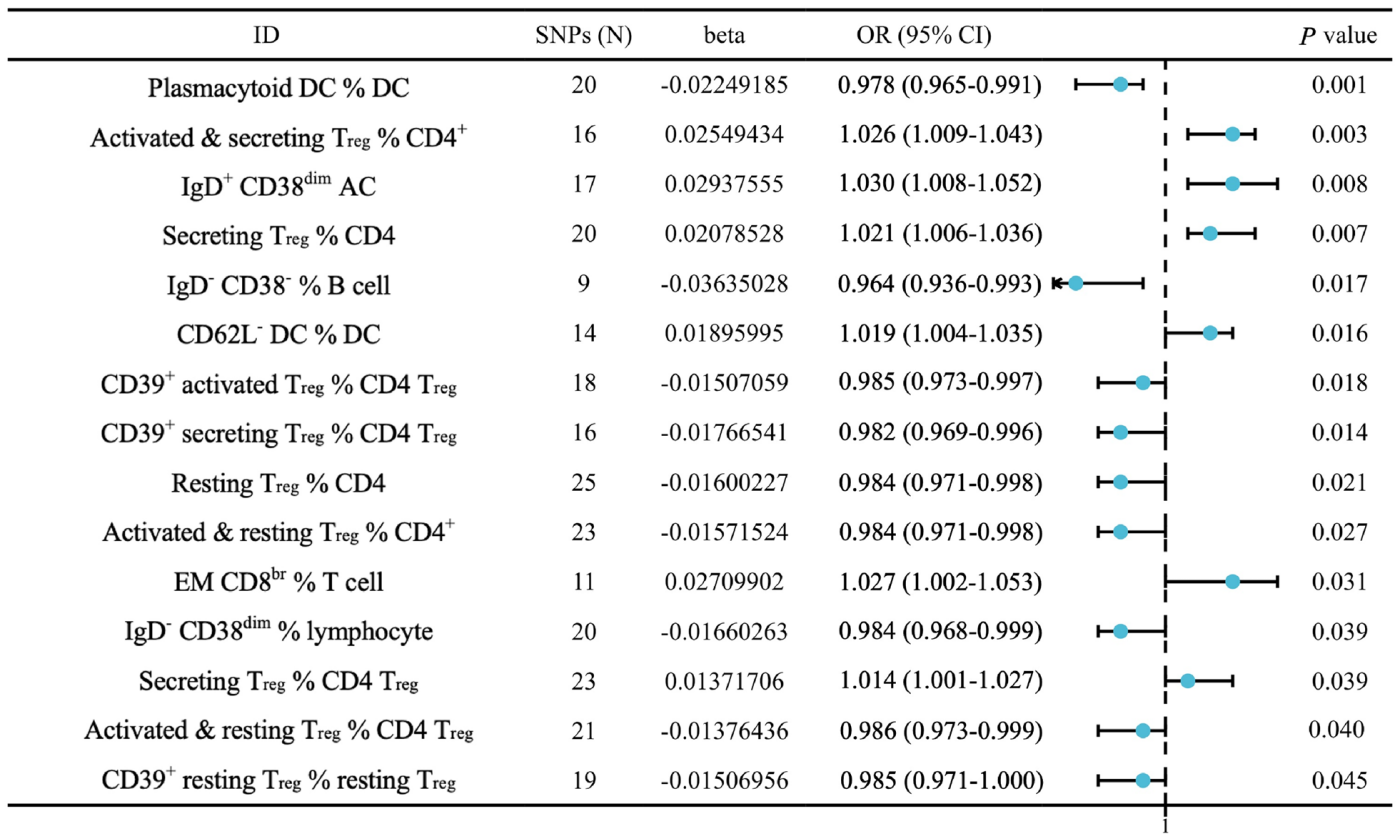
Mendelian randomization analysis of inverse variance weighting method results between immune cell traits and dental caries. Error bars represent 95% confidence intervals. OR > 1 indicates increased caries risk

The results of the genetically predicted IVW method for the seven groups of immune cells against dental caries were shown in Fig. 5. This analysis provided robust causal evidence that specific immune cell subsets directly influence dental caries risk, with DC subsets showing opposing effects. The traits of the following six immune cells (activated & secreting T_reg_ % CD4, IgD^+^ CD38^dim^ AC, secreting T_reg_ % CD4, CD62L^−^DC % DC and secreting T_reg_ % CD4 T_reg_) were positively correlated with dental caries development (OR > 1, *P* < 5.0E-02). Of note, the remaining nine traits (plasmacytoid DC [pDC] % DC, IgD^−^CD38^−^ % B cell, CD39^+^ activated T_reg_ % CD4 T_reg_, CD39^+^ secreting T_reg_ % CD4 T_reg_, resting T_reg_ % CD4, activated & resting T_reg_ % CD4^+^, IgD^−^ CD38^dim^ % lymphocyte, activated & resting T_reg_ % CD4 T_reg_ and CD39^+^ resting T_reg_ % resting T_reg_) reduced the incidence of dental caries (OR < 1, *P* < 5.0E-02). Interestingly, different DC subsets exerted distinct effects on dental caries: CD62L^−^DC increased the risk of dental caries, whereas pDC % DC decreased it. These results provided evidence that CD62L^−^DC activity promotes the occurrence of dental caries (Online Appendix Fig. 6).

Results of the sensitivity analysis confirmed the robustness of our findings. None of the above 15 immunocyte phenotypes for the MR analysis of dental caries showed heterogeneity (*P* > 5.0E-02 for Cochran’s Q test), indicating consistent effects across genetic instruments. The MR-Egger intercept test showed no evidence of horizontal pleiotropy for any of the associations (*P* > 5.0E-02), suggesting that the genetic variants affected dental caries only through their effects on immune cell traits. All IVs had F statistics greater than 10, confirming no weak instrument bias. These results confirm that the credibility of our causal inferences was credible (Online Appendix Table 8).

## Discussion

With the growth in GWAS over the past decades, GWAS has emerged as a powerful tool for identifying genetic variants associated with diseases [19]. Enamel hypoplasia is a cause of dental caries, while dental pulp inflammation is an adverse outcome of progression of dental caries. Epigenetics plays an important role in the development of tooth enamel and also a significant role in the occurrence and progression of dental pulp inflammation. As the most ubiquitous reversible RNA post-transcriptional modification in eukaryotes, m^6^A modification plays an essential role in various diseases [20]. However, there are currently no literature reports indicating a direct relationship between m^6^A modification and the occurrence and development of dental caries.

Our study integrated m^6^A and GWAS data and identified dental caries-associated m^6^A-SNPs for the first time to further elucidate their biological functions and the relationship between m^6^A-SNPs and gene expression (Appendix Fig. 7). M^6^A-related SNPs can affect m^6^A methylation by altering the target site RNA sequence or key flanking nucleotides, thereby exerting biological functions. For instance, SNP rs11266744 participated in oral ulcer pathogenesis by regulating the local expression of *CCRL2* [7]. The functional variant rs2723183 was predicted to affect m^6^A methylation and *IL-37* mRNA expression, as well as alter regulatory motif binding, indicating it was involvement in the pathogenesis of periodontitis [5]. Further, the G allele of rs2259956 altered the m^6^A methylation level of *ABHD12,* which may increase dental caries susceptibility by affecting nearby m^6^A modification sites.

Notably, *ABHD12* was associated with another neurological disorder, PHARC syndrome, which has a clinical picture similar to that of Charcot–Marie–Tooth disease [21, 22]. *ABHD12* encoded a serine hydrolase involved in endocannabinoid metabolism and lysophospholipid signaling [23]. The mechanistic link between *ABHD12* upregulation and caries progression likely involved disruption of endocannabinoid-mediated anti-inflammatory signaling. *ABHD12* specifically hydrolyzed 2-arachidonoylglycerol (2-AG), a major endocannabinoid that normally suppresses inflammatory responses through CB1 and CB2 receptor activation [24]. Elevated *ABHD12* expression in carious pulp accelerated 2-AG degradation, reducing local concentrations of this protective lipid mediator and creating a pro-inflammatory microenvironment [25]. This mechanism established a pathological positive feedback loop where initial bacterial invasion triggers *ABHD12* upregulation, leading to diminished endocannabinoid tone, enhanced inflammatory cell recruitment, and further tissue damage that perpetuates the caries process. This was consistent with our spatial transcriptome data showing *Abhd12* expression in jaw and tooth regions during development, and its presence in phagocytic cells in zebrafish models.

With the recent development of single-cell sequencing technology, the exploration of gene expression patterns of different clusters would help improve our understanding of the underlying mechanisms and their impacts on disease development. Therefore, single-cell sequencing data were comprehensively analyzed to examine dental caries-associated m^6^A-SNP gene expression profiles and elucidate the functional mechanisms of dental caries. UMAP results showed that *ABHD12* was mainly expressed in the carious pulp and was associated with increased dental caries incidence, consistent with the GWAS and cis-eQTL analysis results.

In the single-cell data analysis, DCs account for a relatively large proportion in the carious dental pulp. To investigate the causal relationship between dental caries and immunocyte phenotypes, two-sample MR analysis was used. Evidence of a causal association between CD62L^−^DCs and dental caries was obtained; further, 14 other immunocyte phenotypes were found to be causally associated with dental caries. The m^6^A modifications could influence host defense mechanisms by altering immune cell responses, potentially affecting the oral microbiome composition or the tooth’s resistance to acid demineralization. This gene–environment interaction might explain the variable penetrance of genetic factors in different populations with varying dietary habits, oral hygiene practices, and fluoride exposure levels.

Gene enrichment analysis identified oxidative phosphorylation as a key pathway, consistent with previous studies showing its enrichment in the saliva from children with early childhood caries and its activation in inflamed dental pulp [26, 27]. While the mechanisms linking oxidative phosphorylation to caries remain unclear, m^6^A modification has been shown to regulate this pathway in other diseases [28, 29]. We hypothesize that the m^6^A-SNP rs2259956 may activate oxidative phosphorylation by altering DC abundance, thereby promoting caries progression. This hypothesis requires further experimental validation.

## Study strengths and limitations

Our study had several strengths, including the integration of multiple cutting-edge approaches (GWAS, m^6^A-QTL, single-cell RNA seq, and MR analysis), the use of large sample sizes (161,113 cases and 216,164 controls), and the functional validation through single-cell transcriptomics. In this study, *ABHD12* was identified and characterized as a novel m^6^A-regulated target in dental caries. Mechanistically, we provided suggestive evidence for the causal relationship between multiple immune phenotypes and dental caries, and also propose the hypothesis that DCs interact with dental caries through oxidative phosphorylation.

However, several limitations should be acknowledged: (a) Population specificity: As discussed above, the exclusive use of Finnish GWAS data limits generalizability; (b) Temporal dynamics: Our single-cell data represent snapshots of healthy and carious pulp and do not capture the dynamic progression of caries development; (c) Functional validation: While this study demonstrated associations and expression changes, direct functional experiments confirming the role of m^6^A modification of *ABHD12* in caries development are needed; (d) Environmental factors: Our genetic approach does not fully account for important environmental factors, such as sugar consumption, oral microbiome composition, and access to preventive care. To enhance the global applicability of these findings, we recommend replication studies in diverse populations (particularly those with a high caries burden), development of population-specific polygenic risk scores for dental caries, investigation of gene-environment interactions across different cultural and dietary contexts.

## Conclusion

In summary, this study revealed for the first time that m^6^A modification may be involved in the pathogenesis of dental caries. Bioinformatic analysis identified rs2259956 was associated with the m^6^A modification of the *ABHD12* gene, and this altered m^6^A modification can regulate *ABHD12* expression. The upregulation of *ABHD12* could alter the function of DCs and the process of oxidative phosphorylation, shift dental pulp immunity toward a pro-inflammatory state, and thereby increase the risk of dental caries.

## Supplementary Information

Supplementary material 1.

Supplementary material 2.

Supplementary material 3.

## Data Availability

The data of this study are available from the corresponding author upon reasonable request.

## Abbreviations

GWAS: Genome-wide association studies
m^6^A: *N*^6^-Methyladenosine
m^6^A-SNPs: M^6^A single-nucleotide polymorphisms
MR: Mendelian randomization
m^6^A-QTL: M^6^A quantitative trait loci
PheWAS: Phenome-wide association analysis
hpf: H post-fertilization
MeRIP-seq: Methylated RNA immunoprecipitation sequencing
eQTL: Expression quantitative trait locus
sQTL: Splicing quantitative trait loci
UMAP: Uniform manifold approximation and projection
GO: Gene Ontology
GSEA: Gene-set enrichment analysis
FDR: False discovery rate
SD: Standard deviations
OR: Odds ratios
CI: Confidence intervals
LD: Linkage disequilibrium
IVW: Inverse-variance weighted
DC: Dendritic cell
BP: Biological processes
CC: Cellular components
MF: Molecular functions
TF: Transcription factor
FC: Fold-change
pDCs: Plasmacytoid DCs
