# Supplementary material for: Characterization of m6A-regulated targets and immune cells in dental caries: insights from multi-omics analysis
Source: Eur J Med Res. 2025 Dec 24;30:1258. doi: 10.1186/s40001-025-03514-2 (PMC12729324; doi:10.1186/s40001-025-03514-2)
Supplement: Supplementary file 1 — Supplementary material 1. [file 40001_2025_3514_MOESM1_ESM.docx]

**Characterization of m^6^A-regulated targets and immune cells in dental caries**

Xi Zhou^b,c†^, Cheng Shi^d†^, Kang Li^e,f,g†^, Siyue Yao^a^, Lan Ma^e,f,g^, Yelin Mao^a^, Lingling Jiang^e,f,g^, Pengfei Jiao^a*^

**Affiliations**

^a^The Affiliated Stomatology Hospital of Suzhou Vocational Health College, Suzhou, China

^b^Changsha Stomatological Hospital, Changsha, China

^c^School of Stomatology, Hunan University of Chinese Medicine, Changsha, China.

^d^Nanjing Municipal Center for Disease Control and Prevention, Nanjing, China

^e^The Affiliated Stomatology Hospital of Nanjing Medical University, Nanjing, China

^f^State Key Laboratory Cultivation Base of Research, Prevention and Treatment for Oral Diseases, Nanjing, China

^g^Jiangsu Province Engineering Research Center of Stomatological Translational Medicine, Nanjing, China

^†^These authors contributed equally to this work.

***Corresponding authors**: Pengfei Jiao, The Affiliated Stomatology Hospital of Suzhou Vocational Health College, Suzhou, China. Telephone number: 0512-65163515. Email: hxkqjpf@163.com.

**
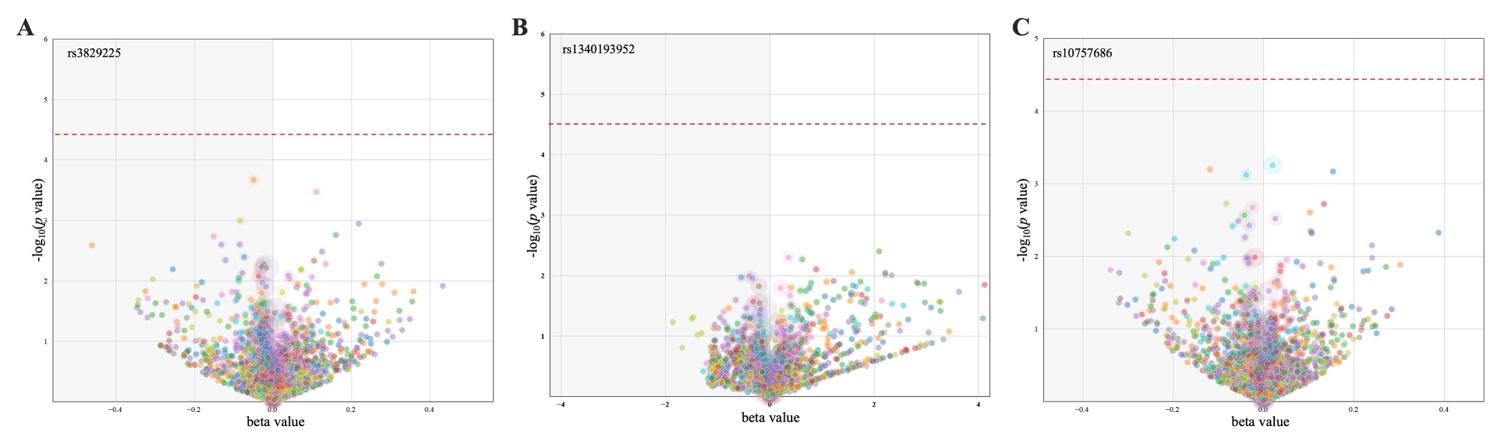
Appendix Figure 1**

**Appendix Figure 1.** A-C. Lavaa Plot of PheWAS for rs3829225, rs1340193952 and rs10757686. This figure represents 2,270 phenotypes tested for association with the SNP. The upper-red line indicates Bonferroni correction *P* = 2.2E-05 (false discovery rate = 0.1 for the entire PheWAS).

**
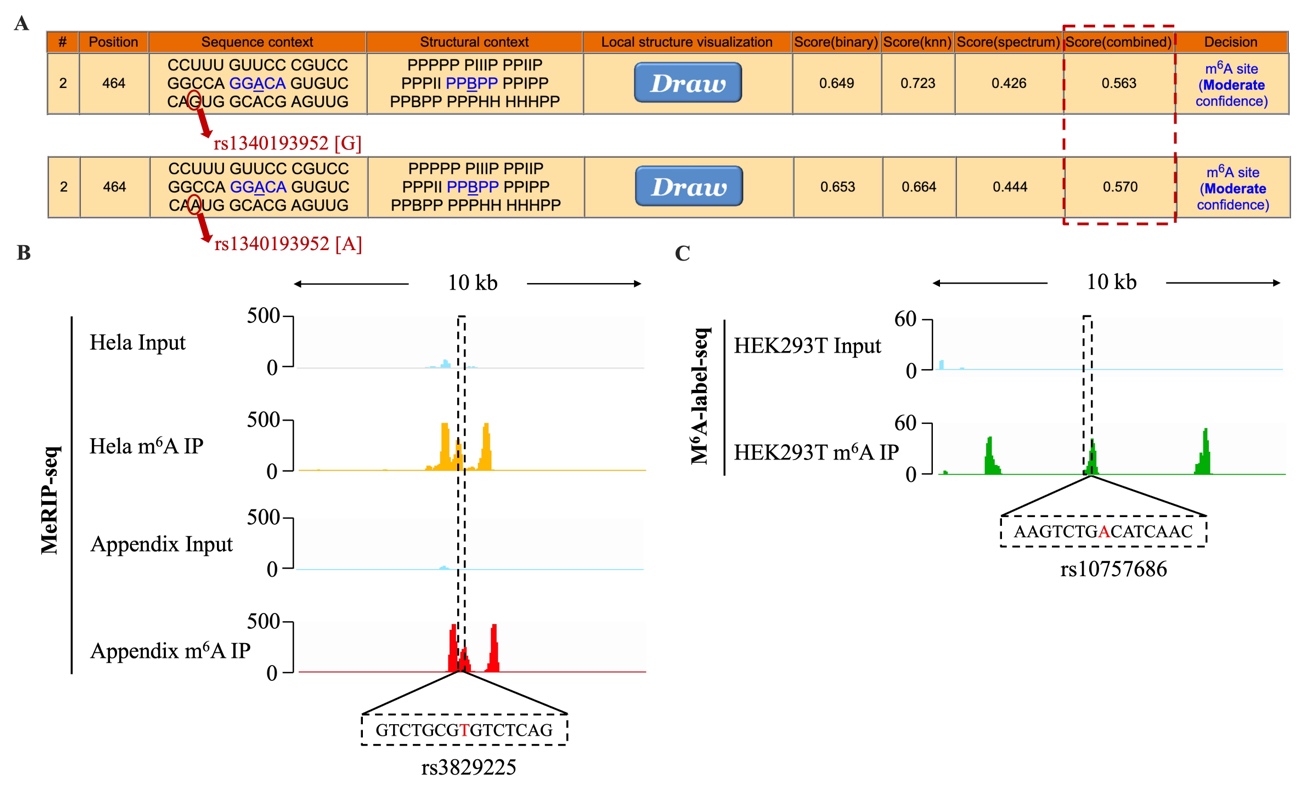
Appendix Figure 2**

**Appendix Figure 2.** A. The rs1340193952 variant located 10 bp from a predicted m^6^A site annotated using the SRAMP database. B. The m^6^A abundances of rs3829225 in Hela and appendix cells compared to controls. C. The m^6^A abundances of rs10757686 in HEK293T cells using m^6^A-label-seq.

**
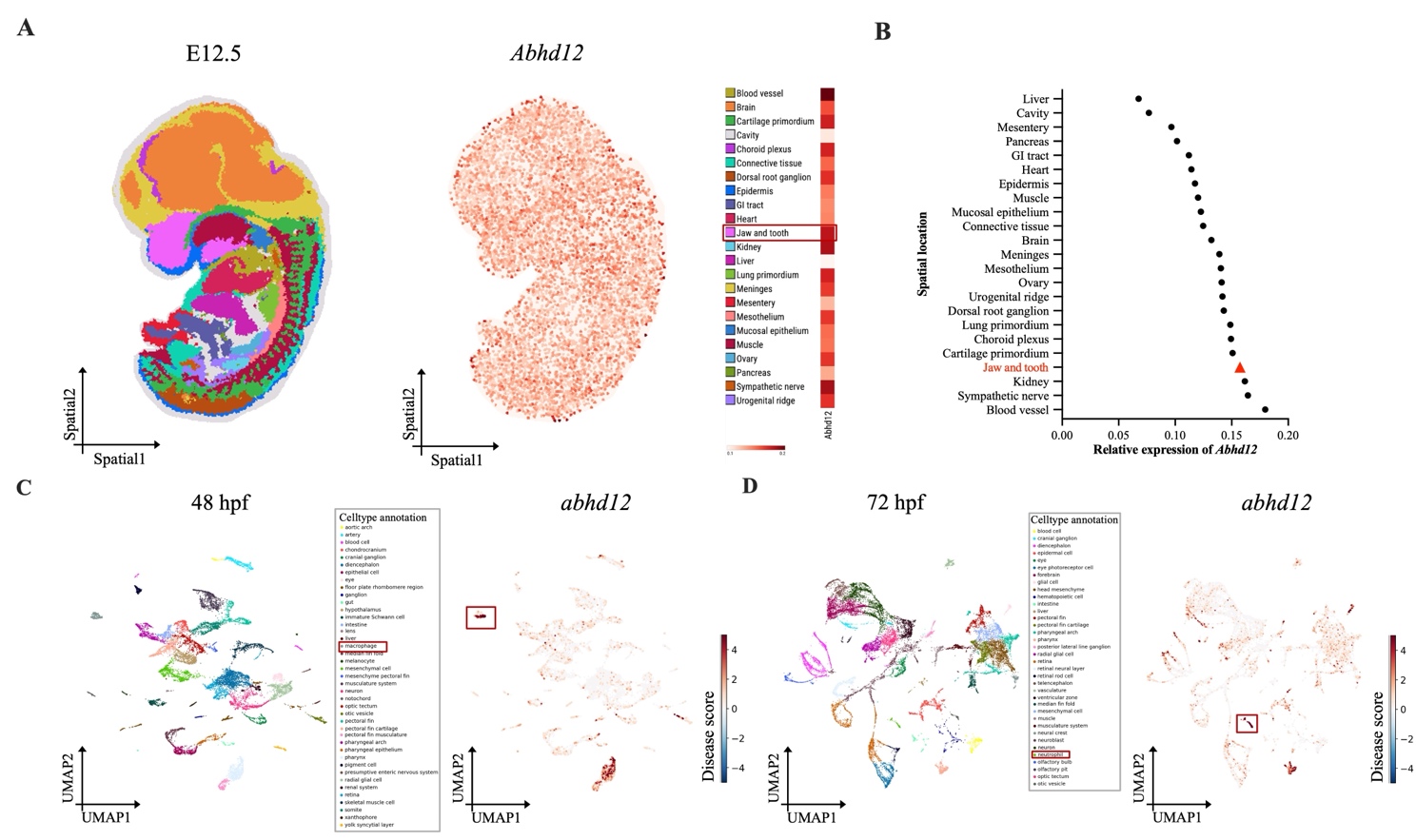
Appendix Figure 3**

**Appendix Figure 3.** A-B. The expression of *Abhd12* gene in mouse embryos of spatial transcriptome data in E12.5. C-D. The expression of *abhd12* gene in zebrafish embryos at 48 and 72 hpf in scRNA-seq data.

**
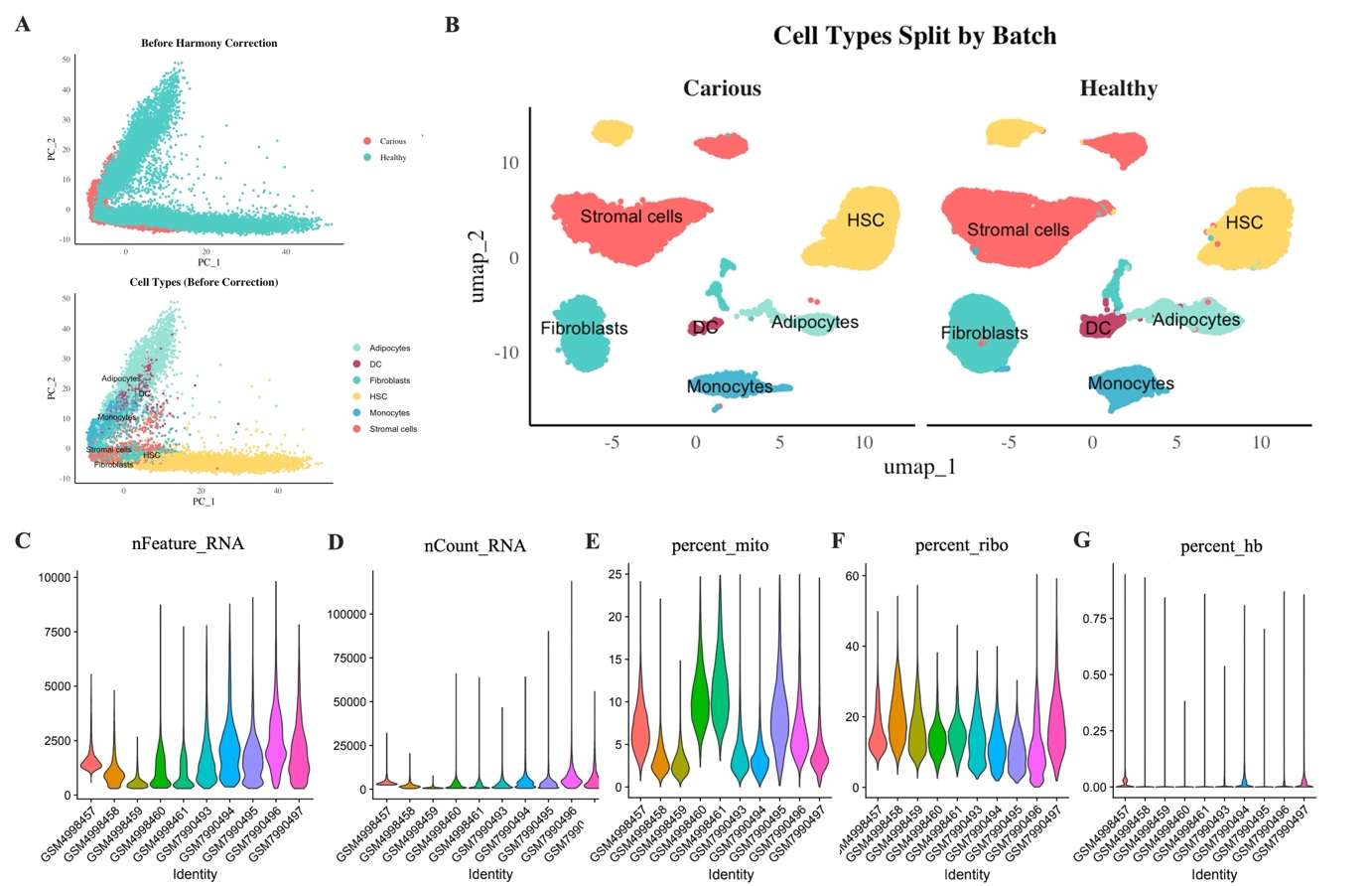
Appendix Figure 4**

**Appendix Figure 4.** A. PCA plots before (top) and after (bottom) Harmony batch correction. Cells are colored by cell type. Correction eliminates batch effects while preserving biological cell type clustering. B. UMAP visualization of cell types split by sample condition (Carious vs. Healthy). Cells colored by identity show consistent clustering patterns across both conditions. C-F. Quality control and data cleaning of sequencing data. The gene data detected in each cell, the total number of molecules detected within the cell, and the proportion of mitochondrial genome in the cell.

**
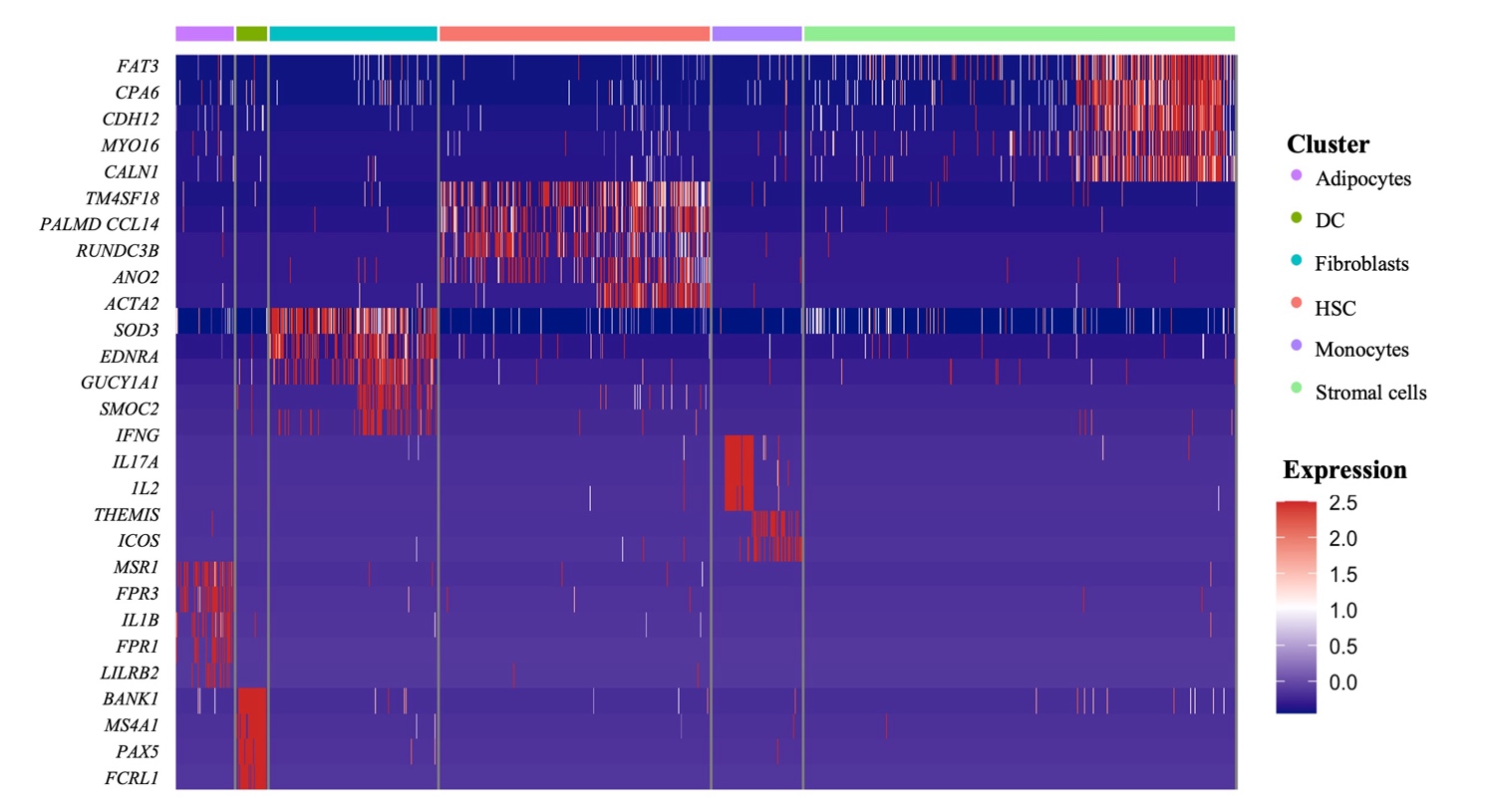
Appendix Figure 5**

**Appendix Figure 5.** Heatmap showing the expression of the top five enriched genes in each cluster.

**Appendix Figure**
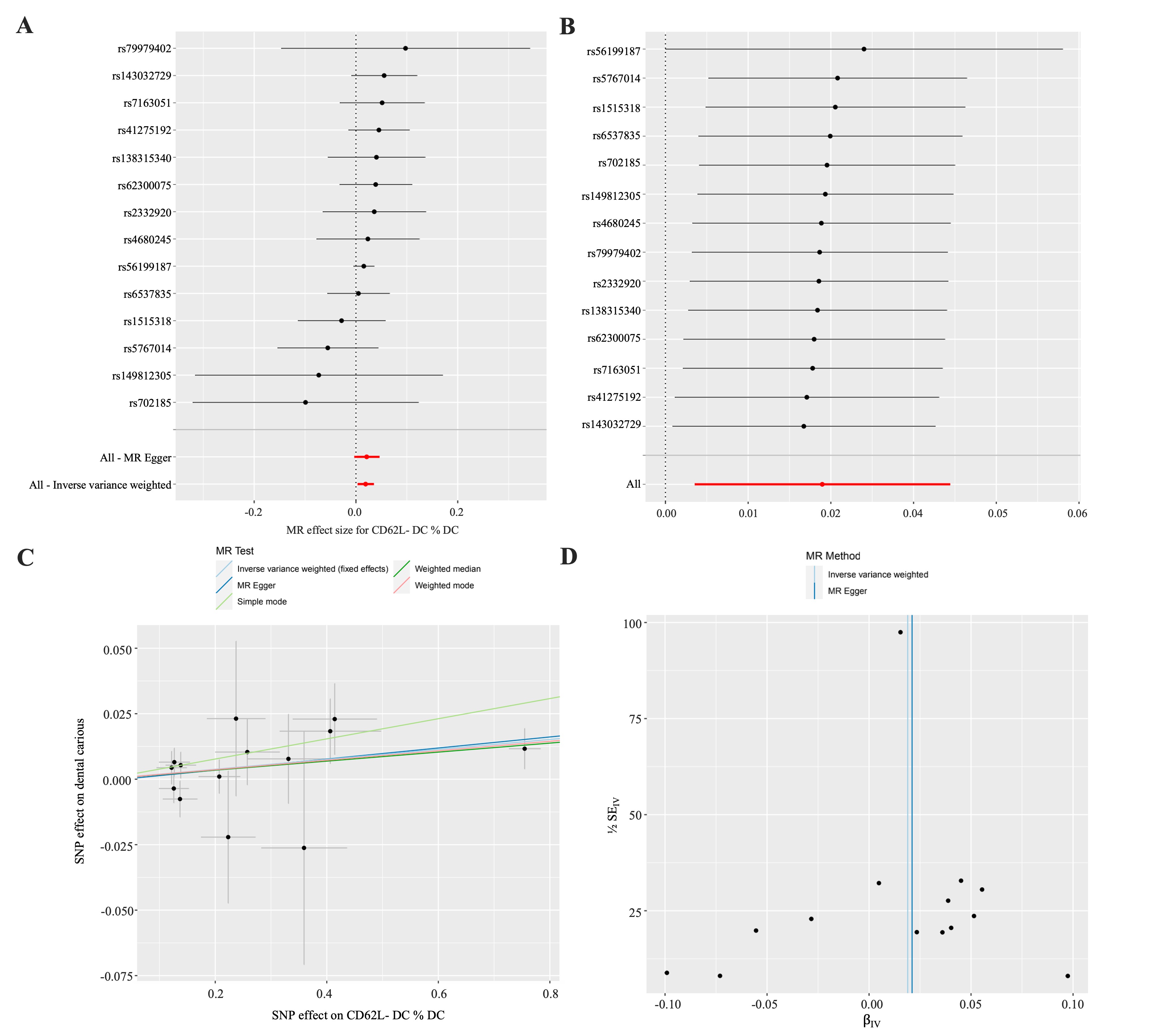
**6**

**Appendix Figure 6.** Result evaluation of Mendelian randomization analysis of CD62L-DC %DC. A. Forest plot. B. Leave-one-out. C. Scatter plot. D. Funnel plot.

**Appendix Figure 7.**


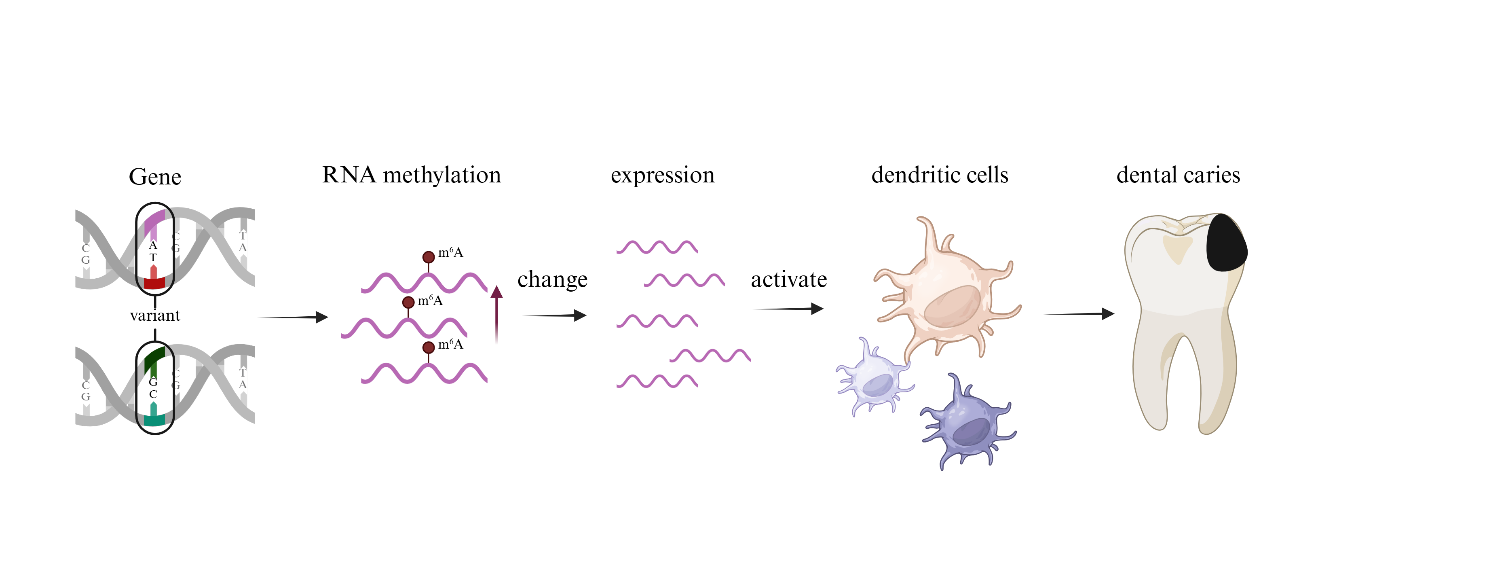


**Appendix Figure 7.** Flowchart of m^6^A-SNP Mechanisms in Dental Caries.

**Appendix Table 1**. Functions and associated diseases of the SNP of interest.

| Gene | Related diseases and function | **References** |
| --- | --- | --- |
| *BRSK2* | Human type 2 diabetes mellitus, developmental delay and intellectual disability, Triple-Negative Breast Cancer, idiopathic pulmonary fibrosis, Autism Spectrum Disorder, pancreatic cancer, autoimmune thyroiditis, paraneoplastic limbic encephalitis, osteoporosis, hepatocellular carcinoma, metastatic osteosarcoma, Primary Squamous Cell Carcinoma, hypertrophic Cardiomyopathy, Rectal Cancer | PMID: 37188647, PMID: 30879638, PMID: 38582395, PMID: 39838395, PMID: 37961520, PMID: 28591720, PMID: 37183696, PMID: 16165222, PMID: 38528539, PMID: 37023992, PMID: 25434727, PMID: 38775423, PMID: 36499607, PMID: 35409691 |
| *IRAG* | a main signal transducer of PKG activity in the cardiovascular system and Digestive system, a non-syndromic form of isolated familial achalasia | PMID: 21666108, PMID: 32573102, PMID：19628652, PMID：34064290 |
| *PUM3* | cell proliferation, Candidate Markers of Olaparib Response, | PMID: 34407138, PMID: 33803939, |
| *ABHD12* | Usher syndrome, Breast Cancer, hepatocellular carcinoma, Neurological Disease, PHARC syndrome, hereditary cataract, Parkinson' s disease, presbycusis, neuroblastoma | PMID: 30974196, PMID: 32366405, PMID: 38053637, PMID: 32364701, PMID: 28448692, PMID: 36891866, PMID: 38750970, PMID: 40055553, PMID: 30978461 |

**Appendix Table 2**. Comparison of m^6^A-SNPs with established caries-associated variants

| Gene | SNP | Chr | Function | Beta/OR | *P*-value | MAF | Mechanism | Reference |
| --- | --- | --- | --- | --- | --- | --- | --- | --- |
| *ABHD12* | rs2259956 | 20 | m^6^A regulation | 0.02 | 2.06E-05 | 0.34 | Immune modulation | This study |
| *AMELX* | rs946252 | X | Missense | 1.28* | 1.12E-07 | 0.09 | Enamel formation | Shaffer *et al*., 2015 |
| *TUFT1* | rs2337360 | 1 | Regulatory | 1.20* | 4.71E-06 | 0.21 | Enamel mineralization | Wang *et al*., 2012 |
| *ENAM* | rs1264848 | 4 | Intronic | 1.18* | 2.41E-05 | 0.42 | Enamel matrix | Zeng *et al*., 2013 |

*Odds ratio reported

**Appendix Table 3**. Integration method quality control metric

| metric | value |
| --- | --- |
| Integration Method | Harmony |
| Reduction Used for QC | harmony |
| Average Silhouette Score | 0.03 |
| Cells from Healthy Batch | 23965 |
| Cells from Carious Batch | 29630 |
| Total Number of Cell Types | 6 |
| Cell Types with Both Batches | 6 |
| Batch Mixing Efficiency | 100% |

**Appendix Table 4**. Statistical comparison of *ABHD12* gene expression between caries and control groups

| Statistical Test | *P* value | Effect size |
| --- | --- | --- |
| Welch's t-test | 1.12E-143 | -0.22 |
| Mann-Whitney U test | 0 | 0.11 |
| Kolmogorov-Smirnov test | 2.30E-155 | NA* |

*NA = not applicable.

**Appendix Table 5**. Differential abundance of cell types between carious and healthy dental tissues

| celltype | Percentage  Carious | Percentage  Healthy | Fold Change | Log2_FC (Healthy/carious) | Odds ratio* | *P* value* | *P* value adj* |
| --- | --- | --- | --- | --- | --- | --- | --- |
| DC | 6.39 | 0.20 | 32.08 | -5.00 | 0.03 (0.02-0.04) | 0 | 0 |
| Stromal cells | 34.09 | 46.92 | 0.73 | 0.46 | 1.71 (1.65-1.77) | 7.59E-199 | 2.28E-198 |
| Adipocytes | 8.26 | 3.29 | 2.51 | -1.33 | 0.38 (0.35-0.41) | 1.61E-138 | 3.22E-138 |
| Monocytes | 10.23 | 7.19 | 1.42 | -0.51 | 0.68 (0.64-0.71) | 1.25E-35 | 1.87E-35 |
| HSC | 24.13 | 27.18 | 0.89 | 0.17 | 1.17 (1.13-1.22) | 1.04E-15 | 1.25E-15 |
| Fibroblasts | 16.90 | 15.22 | 1.11 | -0.15 | 0.88 (0.84-0.92) | 1.40E-07 | 1.40E-07 |

*Statistical comparison of cell type proportions using Fisher's exact test.

*Odds ratio (OR) with 95% confidence intervals indicates the likelihood of each cell type being present in carious versus healthy tissue. *P* values were adjusted for multiple comparisons. Significance levels: ***P < 0.001.

**Appendix Table 6**. Gene Ontology enrichment analysis results for differentially expressed genes

**Appendix Table 7**. Gene Set Enrichment Analysis results

**Appendix Table 8**. Mendelian randomization sensitivity analysis

| Immune trait | Inverse variance weighted | | MR-Egger | |
| --- | --- | --- | --- | --- |
|  | Q | *P* | intercept | *P* |
| Plasmacytoid DC % DC | 22.64 | 0.254 | 0.000 | 0.961 |
| Activated & secreting T_reg_ % CD4^+^ | 18.09 | 0.258 | 0.002 | 0.519 |
| IgD^+^ CD38^dim^ AC | 15.80 | 0.467 | -0.005 | 0.313 |
| Secreting T_reg_ % CD4 | 23.55 | 0.214 | 0.000 | 0.898 |
| IgD^-^ CD38^-^ % B cell | 7.73 | 0.460 | -0.003 | 0.749 |
| CD62L^-^ DC % DC | 8.88 | 0.782 | -0.001 | 0.828 |
| CD39^+^ activated T_reg_ % CD4 T_reg_ | 8.26 | 0.961 | 0.003 | 0.425 |
| CD39^+^ secreting T_reg_ % CD4 T_reg_ | 13.26 | 0.582 | 0.001 | 0.903 |
| Resting T_reg_ % CD4 | 17.58 | 0.823 | -0.008 | 0.055 |
| Activated & resting T_reg_ % CD4^+^ | 25.25 | 0.285 | -0.002 | 0.458 |
| EM CD8^br^ % T cell | 13.24 | 0.211 | 0.000 | 0.985 |
| IgD^-^ CD38^dim^ % lymphocyte | 18.16 | 0.512 | -0.001 | 0.676 |
| Secreting T_reg_ % CD4 T_reg_ | 24.34 | 0.330 | -0.002 | 0.477 |
| Activated & resting T_reg_ % CD4 T_reg_ | 22.92 | 0.293 | 0.002 | 0.576 |
| CD39^+^ resting T_reg_ % resting T_reg_ | 19.77 | 0.346 | 0.004 | 0.300 |
